# Anti-angiogenic drug aggravates the degree of anti-resorptive drug-based medication-related osteonecrosis of the jaw by impairing the proliferation and migration function of gingival fibroblasts

**DOI:** 10.1186/s12903-023-03034-7

**Published:** 2023-05-27

**Authors:** Ning Zhao, Qing-xiang Li, Yi-fei Wang, Qiao Qiao, Hong-yuan Huang, Chuan-bin Guo, Yu-xing Guo

**Affiliations:** 1 Department of Oral and Maxillofacial Surgery, Peking University School and Hospital of Stomatology, 22 Zhongguancun Nandajie Haidian District, Beijing, 100081 PR China; 2 National Clinical Research Center for Oral Diseases, Beijing, 100081 PR China; 3 National Engineering Laboratory for Digital and Material Technology of Stomatology, Beijing, 100081 PR China; 4 Beijing Key Laboratory of Digital Stomatology, Peking University School and Hospital of Stomatology, Beijing, 100081 PR China

**Keywords:** Anti-angiogenic, Anti-resorptive, Medication-related osteonecrosis of the jaw, Gingival fibroblasts, Proliferation and migration

## Abstract

**Background:**

Long-term use of anti-resorptive or anti-angiogenic drugs in cancer patients with odontogenic infections may lead to medication-related osteonecrosis of the jaw (MRONJ). This study investigated whether anti-angiogenic agents aggravate MRONJ occurrence in anti-resorptive-treated patients.

**Methods:**

The clinical stage and jawbone exposure of MRONJ patients caused by different drug regimens were analyzed to ascertain the aggravation effect of anti-angiogenic drugs on anti-resorptive drug-based MRONJ. Next, a periodontitis mice model was established, and tooth extraction was performed after administering anti-resorptive and/or anti-angiogenic drugs; the imaging and histological change of the extraction socket were observed. Moreover, the cell function of gingival fibroblasts was analyzed after the treatment with anti-resorptive and/or anti-angiogenic drugs in order to evaluate their effect on the gingival tissue healing of the extraction socket.

**Results:**

Patients treated with anti-angiogenic and anti-resorptive drugs had an advanced clinical stage and a bigger proportion of necrotic jawbone exposure compared to patients treated with anti-resorptive drugs alone. In vivo study further indicated a greater loss of mucosa tissue coverage above the tooth extraction in mice treated with sunitinib (Suti) + zoledronate (Zole) group (7/10) vs. Zole group (3/10) and Suti group (1/10). Micro-computed tomography (CT) and histological data showed that the new bone formation in the extraction socket was lower in Suti + Zole and Zole groups vs. Suti and control groups. In vitro data showed that the anti-angiogenic drugs had a stronger inhibitory ability on the proliferation and migration function of gingival fibroblasts than anti-resorptive drugs, and the inhibitory effect was obviously enhanced after combining zoledronate and sunitinib.

**Conclusion:**

Our findings provided support for a synergistic contribution of anti-angiogenic drugs to anti-resorptive drugs-based MRONJ. Importantly, the present study revealed that anti-angiogenic drugs alone do not induce severe MRONJ but aggravate the degree of MRONJ via the enhanced inhibitory function of gingival fibroblasts based on anti-resorptive drugs.

**Supplementary Information:**

The online version contains supplementary material available at 10.1186/s12903-023-03034-7.

## Background

Medication-related osteonecrosis of the jaw (MRONJ) is a rare but potentially serious complication caused by anti-resorptive and anti-angiogenic agents [1]. If left untreated, it can lead to chronic infection, pain, and oral dysfunction. It can also change the facial appearance, thus substantially decreasing the patients’ quality of life [2]. The most common anti-resorptive drugs associated with MRONJ are bisphosphonates, denosumab, and romosozumab [3], which are commonly used to treat cancer and osteoporosis. Yet, the incidence of MRONJ seems to be higher in cancer patients treated with high-potency bisphosphonates or denosumab (it can reach 7%) and lower (0.01%-0.1%) in osteoporosis patients using low-potency drugs [4]. Also, more recently, a strong association between MRONJ and anti-angiogenic drugs has been found [5].

Angiogenesis, the process leading to the formation of new blood vessels, is one of the hallmarks of cancer. Anti-angiogenic drugs that target vascular endothelial growth factors (VEGF)/VEGF receptor signaling have been widely used in the treatment of malignant tumors, such as metastatic renal cancer, lung cancer, breast cancer, etc. [6]. Anti-angiogenic inhibitors can be divided into three categories, i.e., anti-VEGF monoclonal antibody (e.g., bevacizumab), VEGF decoy receptors or VEGF-Trap (e.g., aflibercept), and small molecule tyrosine kinase inhibitors (TKI) that block the VEGF receptors downstream signaling pathways (e.g., sunitinib, cabozantinib, and sorafenib [4]. Some studies suggested that the combined use of anti-angiogenic and anti-resorptive drugs can aggravate the disease severity of MRONJ [7]. Guarneri et al*.* revealed a 30-fold increase in the incidence of MRONJ in patients who received bevacizumab with concomitant bisphosphonate therapy compared to bevacizumab alone (1.8%, 12/658 vs. 0.06%, 2/2902 with bevacizumab alone) [8]. In addition to the anti-angiogenic effects of anti-angiogenic inhibitors, anti-resorptive drugs have also been reported to have anti-angiogenic effects during the development of MRONJ [9, 10]. Therefore, the role of the two types of drugs in the pathogenesis of MRONJ needs to be further clarified, as this might be beneficial to prevent the occurrence of MRONJ or guide the adjustment of drug regimens during MRONJ treatment.

Based on such recognition, we hypothesized that the use of anti-angiogenic agents aggravates MRONJ occurrence due to impaired mucosal healing in the anti-resorptive-treated patients, leading to the exacerbation of clinical staging and increase of bone exposure chances.

## Methods

### Patients

Patients diagnosed with MRONJ who were previously treated with anti-resorptive therapy alone, or combined with anti-angiogenic therapy in the Department of Oral and Maxillofacial Surgery, Peking University School and Hospital of Stomatology (PKUSHS) between January 2015 and December 2021 were enrolled in this retrospective study. The inclusion criteria were: (1) MRONJ was diagnosed based on the AAOMS (American Association of Oral and Maxillofacial Surgeons) definition to distinguish it from any other delayed healing conditions [1]; (2) received treatment with anti-resorptive or anti-angiogenic agents; (3) exposed bone or bone that can be probed through an intraoral or extraoral fistula in the maxillofacial region, which persisted for longer than 8 weeks; (4) no history of radiation therapy to the jaws or obvious metastatic disease to the jaws. Exclusion criteria: patients who did not complete imaging or did not complete treatment, or failed to undergo regular follow-up.

The study involved human data, which was approved by the Institutional Biomedicine Ethics Committee of Peking University Hospital of Stomatology (PKUSSIRB-201949114). All the participants provided written informed consent. All experimental procedures were performed in accordance with the Declaration of Helsinki.

### Animal model

Forty male C57BL6/N mice, 6-8 weeks-old and weighing 25 ± 5 g, were purchased from Vital River Laboratory Animal Technology (Beijing). All the animals were housed in an environment with a temperature of 22 ± 1 °C, relative humidity of 50 ± 1%, and a light/dark cycle of 12/12 h and received water and food ad libitum. All animal experiments (including the mice euthanasia procedure) were approved by the Ethics Committee of the Peking University Health Science Center (LA2022483) and carried out in compliance with ARRIVE (Animal Research: Reporting of in vivo Experiments) guidelines. All animal experiments and care were carried out in accordance with the Guidelines for the Care and Use of Laboratory Animals.

MRONJ was induced in accordance with the methods described by previous studies [11, 12]. To imitate the conditions that occur in MRONJ-like disease, we placed sterile silk 5.0 ligature around the left maxillary second molar in a submarginal position to induce periodontitis at the beginning of the administration of the above drugs [13]. Mice were then randomly assigned to the following four groups (Table S1): control group (Ctrl), zoledronate group (Zole), sunitinib group (Suti), and zoledronate + sunitinib group (Zole + Suti). Mice in the zoledronate group was intraperitoneally administered with zoledronate (SML0223, sigma) at 1 mg/kg of body weight every other day; mice in the sunitinib group received sunitinib (S7781, Selleck Chemicals) orally at 40 mg/kg of body weight every other day; mice in the zoledronate + sunitinib group received both of the two drugs; and mice in the control group received the same amount of saline and corn oil. Drugs were administered for two weeks before the left maxillary second molar extraction. And then, the same tooth was atraumatically extracted under general anesthesia. After extraction, drugs were continuously used for another two weeks.

Mice were then euthanized by cervical dislocation, and maxillary bones were retrieved. The specimens were photographed by a stereoscopic microscope, fixed in 4% paraformaldehyde (PFA) solution at 20 °C for 24 h, analyzed by micro-computed tomography (micro-CT), decalcified in 15% EDTA solution at 37 °C for 2 weeks, and then embedded in paraffin for further research.

### Incomplete healing evaluation

Incomplete healing was defined as thinner gingival tissue above the extraction socket than the level of the surrounding mucosa, showing a pit-like appearance [14].

### micro-CT

The mice were sacrificed two weeks after the teeth extraction, and the maxillary bone was scanned using a micro-CT (Inveon MM Gantry-STD). The following parameters were applied: at 10-µm resolution with exposure time 1500 ms, electric voltage 60 kV, and current 220 µA. To evaluate bone healing after surgery, the region of interest (ROI) covered the whole extraction socket without root left after exodontia. Quantitative analyses of the ROI were performed using Inveon Research Workplace (Siemens) to evaluate bone mineral density (BMD). Besides, images of the sagittal section and three-dimensional reconstruction were obtained to observe the microstructure change of the extraction socket.

### Histological analysis

The above-embedded specimens were cut into 4 μm thick sections and subjected to H&E (hematoxylin and eosin) and Masson (G1340; Solarbio) staining for the extraction socket evaluation under a light microscope (Olympus, Japan). To quantify the degree of osteonecrosis, three square areas measuring 0.1 mm^2^ were randomly selected as ROI from the alveolar bone surrounding the extraction socket in each animal, and the average number of empty lacunae per 0.1 mm^2^ was counted using ImageJ software. In addition, a TRAP (Tartrate resistant acid phosphatase) staining kit (387A; Sigma-Aldrich) was used to detect TRAP^+^ osteoclasts according to the manufacturer’s instructions, and the average number of osteoclasts was counted by using a previously described approach [12].

### Cell culture and treatment

The human gingival fibroblasts (HGFs) were obtained from the central laboratory of Peking University School and Hospital of Stomatology. The cells were cultured in DMEM (Gibco, USA) containing 10% FBS (Sigma, USA) and 1% penicillin/streptomycin in a humidified atmosphere containing 5%CO_2_/95% air at 37ºC. To detect the effect of MRONJ-related drugs on HGFs, sunitinib (10μΜ) or zoledronate (5μΜ) alone or sunitinib (10μΜ) + zoledronate (5μΜ) was added to the culture medium.

### Colony formation assay

One hundred cells were seeded into 6-well plates and treated with drugs (as explained above; see section Cell culture and treatment) for 10 days. Next, cells were first fixed in 4% PFA solution for 10 min, rinsed in PBS, then stained with 0.1% crystal violet and photographed, after which they were counted. Besides, each group's colonies' images were captured by an inverted fluorescence microscope.

### Cell proliferation assay

HGFs were plated at a density of 3200 cells/well in a 96-well plate and treated with the drugs above (as explained above; see section Cell culture and treatment) for 0, 1, 2, 3, 4, 5, 6, and 7 days. At each time point, cell counting kit-8 (CCK-8) solution (10μL) was added to each well and incubated for another 2 h at 37 °C. The absorbance at 450 nm was determined by using a microplate reader (Thermo Fisher Scientific, USA).

### Wound healing assay

Six-well plates were used to culture HGFs in accordance with the methods and treatment described above. After the cell reached 90% confluence, a line was drawn using a marker on the bottom of the dish, after which a sterile 200-μl pipet tip was used to scratch three separate wounds through the cells, moving perpendicular to the line. The cells were gently rinsed twice with PBS to remove floating cells and incubated culture medium containing drugs (as explained above; see section Cell culture and treatment). Images of the scratches were taken by using an inverted microscope at × 10 magnification at 0 h and 36 h of incubation and analyzed using ImageJ software.

### Cell cycle analysis

The HGFs were seeded in a 6-well plate at a density of 1 × 10^6^ cells/well and treated with drugs (as explained above; see section Cell culture and treatment) for 48 h. The cells were then harvested, washed with PBS, fixed in 70% ethanol, and stored at -20 °C for 4 h. After incubation, cells were re-suspended in PI staining solution and further incubated for 30 min at 37 °C in the dark. The total DNA content was measured by a flow cytometer (DxP Athena, Cytexbio).

### Sample size and statistical analysis

GraphPad Prism 7 software was used for statistical analysis. Two-sided Student’s *T*-tests or analysis of variance were used for statistical analysis to determine significance. A* P*-value < 0.05 was considered significant. N = 10 for all experiments if not stated otherwise. For quantifications of H&E, Masson, and TRAP staining experiments, at least five sections per mouse were examined for comparison. Statistical data were presented as the mean ± SD.

## Results

### The combined use of anti-resorptive drugs and anti-angiogenic drugs upgrades MRONJ staging and increases the proportion of jawbone exposure

In order to ascertain the aggravation effect of the anti-angiogenic drugs on anti-resorptive drug-based MRONJ, we reviewed the clinical data of 105 MRONJ patients and classified them according to the medication regimen. Seventy-four patients were treated with anti-resorptive drugs alone and 31 with anti-resorptive + anti-angiogenic drugs (combined group). There were 20.27% (15/74) patients with stage 3 in the anti-resorptive drug group vs. 41.94% (13/31) patients with stage 3 in the combined group (*P* < 0.05) (Table 1).

**Table 1:** Basic information on the surgical treatment results of MRONJ patients caused by anti-resorptive drugs and anti-resorptive drugs plus anti-angiogenic drugs

**Variable**	**Anti-resorptivedrug group (*n* = 74)**	**Anti-resorptive drug + anti-angiogenic drug group (*n* = 31)**	***P***
Gender			
Male	28 (37.84)	21 (67.74)	0.006^‡^
Female	46 (62.16)	10 (32.26)	
Average age ± SD (year)	65.18 ± 10.34	61.32 ± 5.92	0.061*
Duration of anti-resorptive drugs in months ± SD	37.09 ± 28.91	26.61 ± 15.25	0.005*
Anti-angiogenic medications			
Suntinib	NA	9 (29.03)	NA
Bevacizumab		10 (32.26)	
Anlotinib		5 (16.13)	
others		7 (22.58)	
Underlying disease			
Osteoporosis	11 (14.86)	0 (0.00)	NA
Breast cancer	25 (33.78)	4 (12.90)	
Lung cancer	15 (20.27)	17 (54.84)	
Kidney cancer	0 (0.00)	8 (25.81)	
Prostate cancer	15 (20.27)	0 (0.00)	
Multiple myeloma	7 (9.46)	0 (0.00)	
Others	1 (1.35)	2 (6.45)	
MRONJ location			
Maxilla	24 (32.43)	11 (35.48)	0.466^‡^
Mandible	50 (67.57)	20 (64.52)	
Clinical symptom			
Jaw exposure	31 (41.89)	22(70.97)	0.010
Skin or mucosa fistula	43 (58.11)	9 (29.03)	
MRONJ stage			
1	16 (21.62)	2 (6.45)	0.031^‡^
2	43 (58.11)	16 (51.61)	
3	15 (20.27)	13 (41.94)	
Surgical methods			
Marginal osteotomy	64 (86.49)	24 (77.42)	0.259^‡^
Segment osteotomy	10 (13.51)	7 (22.58)	
Treatment results			
Healed	52 (70.27)	12 (38.71)	0.004^‡^
Non-healing	22 (29.73)	19 (61.29)	

Data presented as n (%), unless otherwise noted. MRONJ, medication-related osteonecrosis of the jaw; SD, standard deviation

^*^ Independent-sample t-test

^‡^χ^2^ test

Exposed jaws and soft tissue fistulas are typical symptoms of MRONJ. The proportion of exposed jaws was 41.89% (31/74) in the anti-resorptive drug group, and 70.97% (22/31) in the combined group. These data suggest that MRONJ patients who receive anti-resorptive and anti-angiogenic drugs are prone to jawbone exposure and escalation of clinical staging.

### Combined use of anti-angiogenic and anti-resorptive drugs can significantly interfere with the mucosal healing ability of the extraction socket

**Fig. 1:**
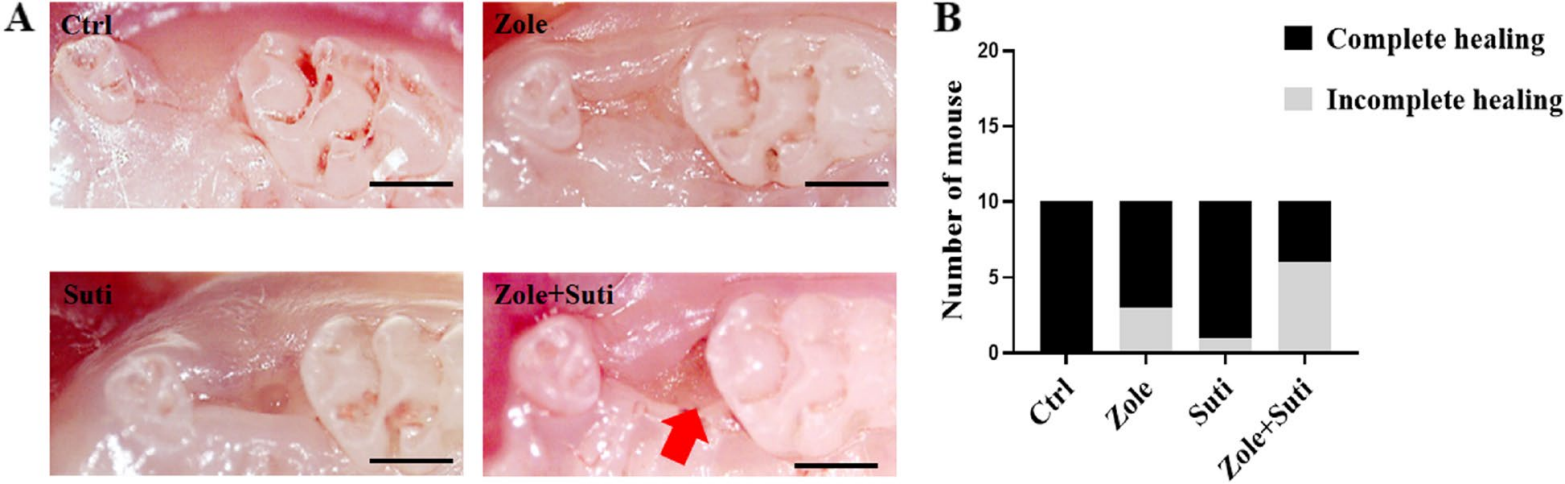
Stereoscopic observation of socket wound healing after tooth extraction. **A** The healing state of the mucosa on the surface of the extraction socket. The red arrow shows the obvious incomplete healing of the extraction socket after the combined drug treatment (Zole + Suti). **B** Statistics of healing status of extraction sockets post different drug treatments. Ctrl, control; Suti, sunitinib; Zole, zoledronic acid. Scale bar in A, 500 μm

In order to mimic the odontogenic infection situation in which a cancer patient needs to have teeth extracted, we first established a periodontitis model of the maxillary second molar (Figure S1). Two weeks after drug administration (zoledronic acid, sunitinib, or a combination of the two), the left maxillary second molars were extracted, and the animals were sacrificed for observation after another two weeks. Stereoscopic observation of tooth extraction wound healing showed that mucosa tissue coverage above the tooth extraction site in the control group was completely healed (10/10). In mice treated with zoledronic acid, the incidence of incomplete mucosal healing was about 30% (3/10), which was higher than that of the mice in the Suti group (10%, 1/10) (Fig. 1). However, in mice treated with Zole + Suti, the incidence of incomplete mucosal healing reached 70% (7/10) (Fig. 1), which showed that the anti-angiogenic drugs alone have a weak effect on the mucosal healing of extraction socket, but once combined with anti-resorptive drugs, the inhibitory effect can be significantly enhanced.

### Combined use of anti-angiogenic and anti-resorptive drugs aggravates osteonecrosis by inhibiting mucosal healing

**Fig. 2:**
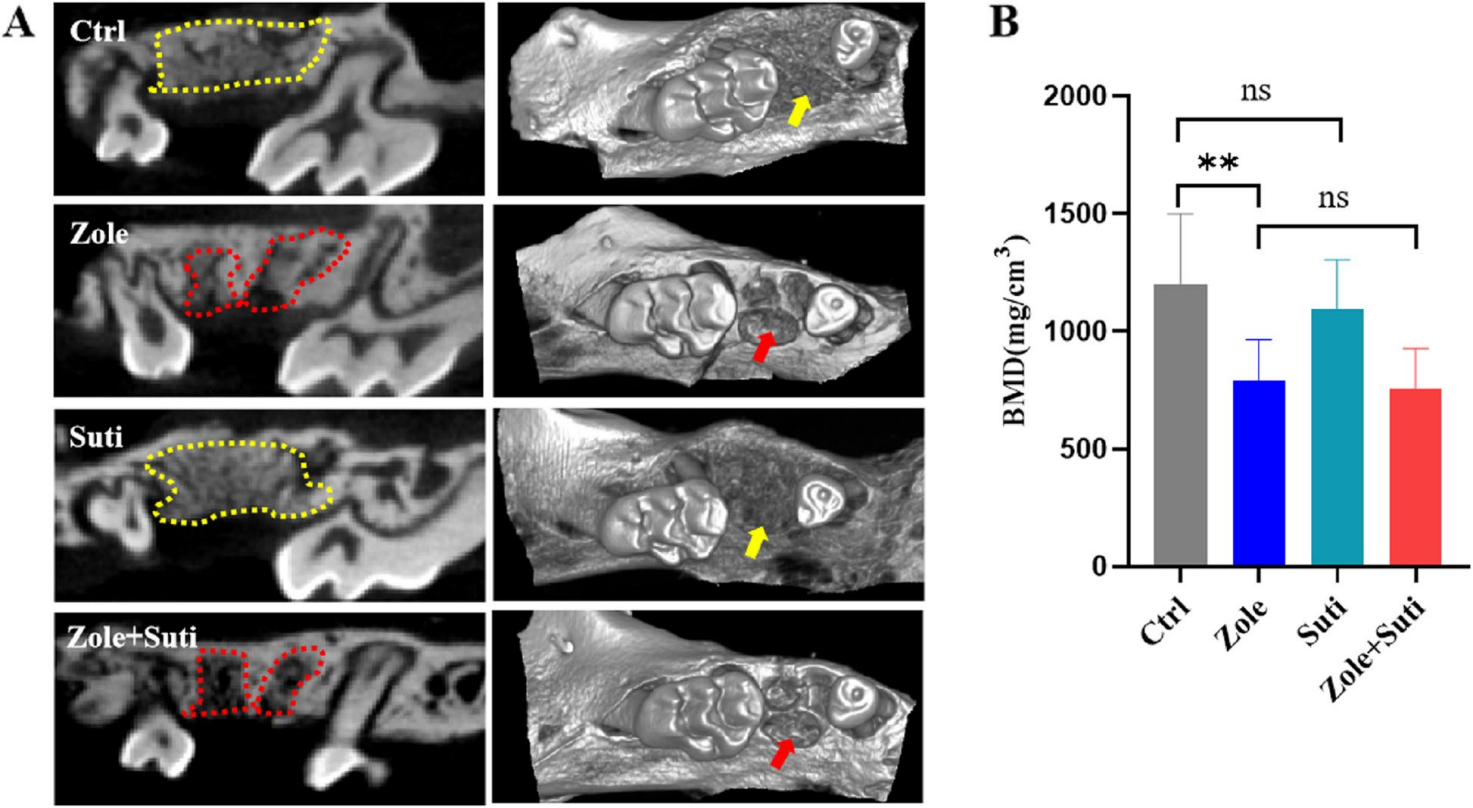
Interference of new alveolar bone formation and alveolar bone remodeling activity in extraction sockets after application of anti-resorptive drugs. **A** Coronal section and 3D reconstruction of micro-CT images of the maxillary bone after two weeks after the left maxillary second molar extraction. The positions pointed by the yellow dotted line and arrow indicate that the new formed alveolar bone of the extracted tooth is well remodeled, while the red dotted lines and arrows is labeled that the socket bone of the extracted tooth is poorly remodeled. **B** Statistical analysis of bone mineral density of newly formed alveolar bone in extraction socket. BMD, bone mineral density

**Fig. 3:**
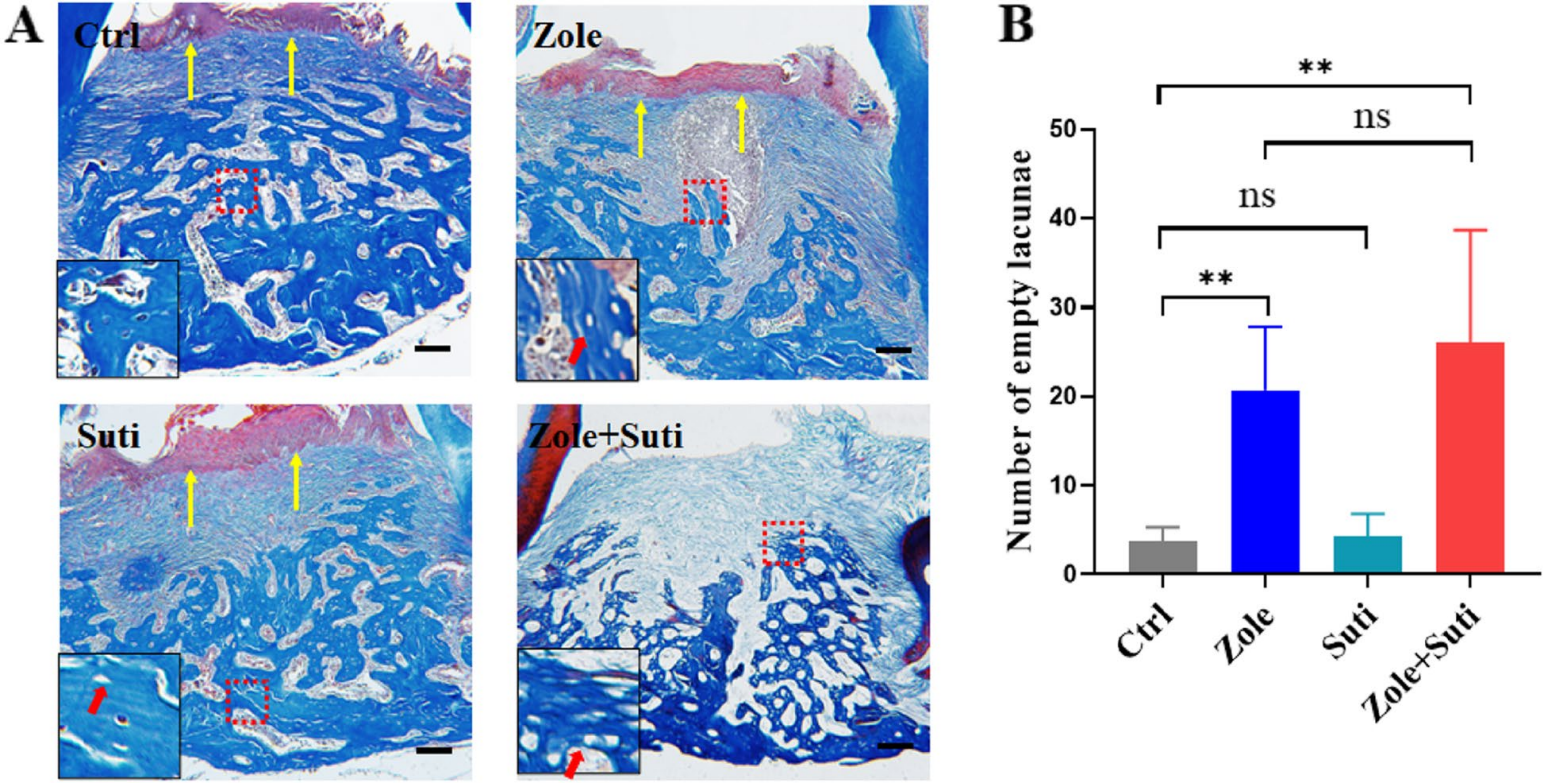
Combined use of anti-resorptive and anti-angiogenic drugs interferes with both soft tissue coverage of extraction sockets and new bone formation in extraction sockets. **A** Masson staining to observe the results of soft tissue coverage and new bone formation in the extraction socket of the left maxillary second molar. The inset shows the boxed region magnified. **B** Statistical analysis of empty bone lacuna in extraction socket post different drug treatments. Yellow arrows indicate the formation of a normal mucosal coverage. Red arrows indicate empty bone lacuna. Scale bar in A, 100 μm

The healing of the extraction socket requires both mucosal coverage and new alveolar bone formation. The micro-CT image analysis revealed that compared with the control group mice, there was no significant difference in the new bone formation in the Suti group mice (Fig. 2). However, in the Zole group and the Zole + Suti group, the amount of new bone formation in the extraction socket was poor, and the extraction socket had a clear boundary outline (Fig. 2). Furthermore, Masson staining of tissue further confirmed these data (Fig. 3A). Meanwhile, the number of empty lacunae was seen in the Zole and Zole + Suti groups, being significantly more abundant than in the control group and the Suti group (*P* < 0.01) (Fig. 3B). In addition, impairment of soft tissue coverage above the extraction socket was obvious in the Zole + Suti group and was not seen in the other three groups (Fig. 3A). The typical epithelial basement membrane structure was lost after combining anti-resorptive and anti-angiogenic drugs (Figure S2). These data suggest that anti-angiogenic drugs may aggravate the degree of MRONJ by inhibiting the healing function of soft epithelial tissue.

### Anti-angiogenic drugs have a stronger inhibitory ability on the colony formation, proliferation, and migration of gingival fibroblasts than anti-resorptive drugs

**Fig. 4:**
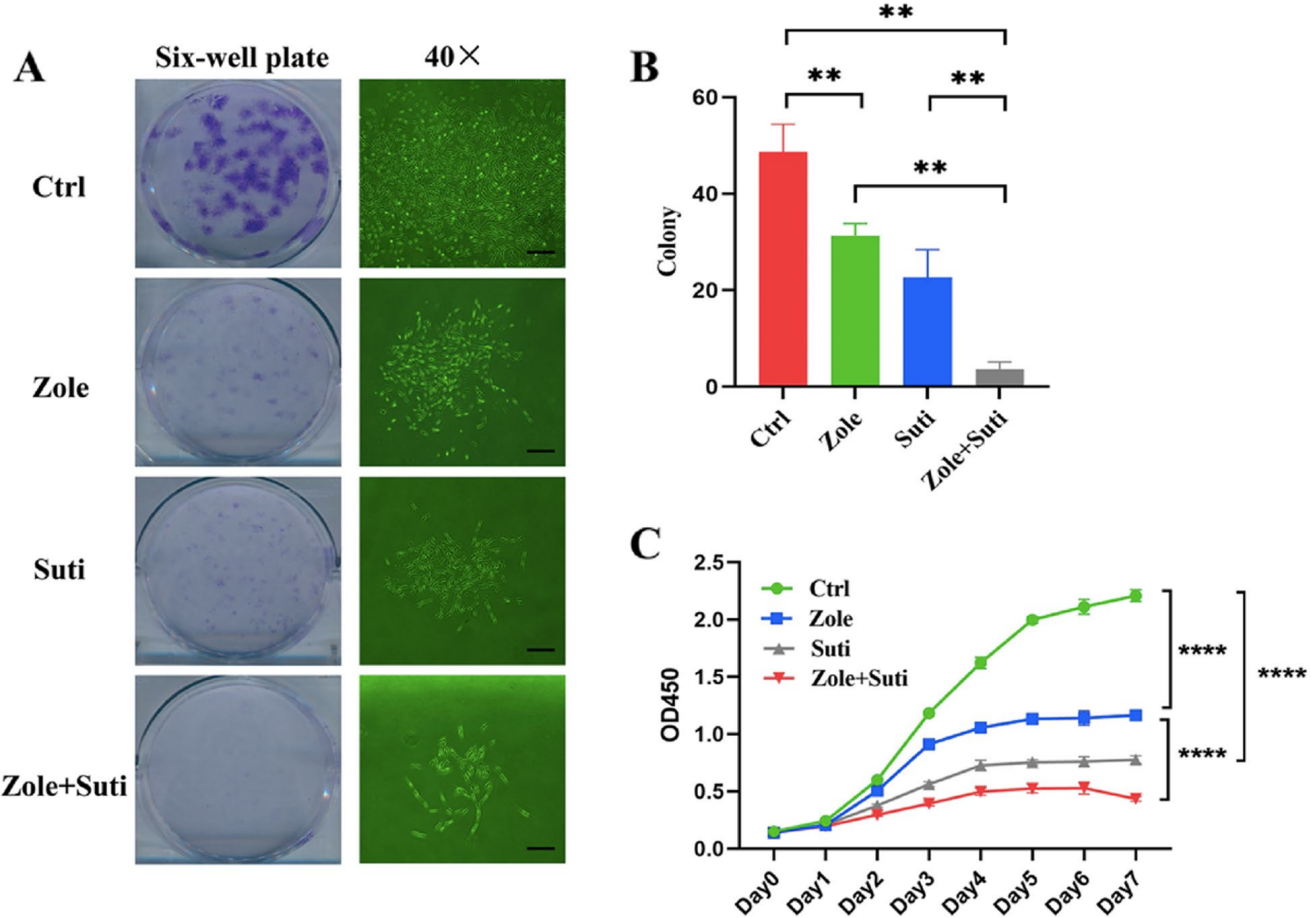
Anti-angiogenic drugs significantly inhibit the clonogenic and proliferative abilities of gingival fibroblasts than anti-resorptive drugs. **A** Crystal violet staining and 40 × microscope observation of 6-well plates with gingival fibroblasts seeded to observe the colony formation ability. **B** Statistical analysis of colony formation numbers of gingival fibroblasts post different drug treatments. **C** Statistical analysis of gingival fibroblasts post different drug treatments using CCK-8 assay. ANOVA was performed. *P < 0.05; **P < 0.01. Scale bars in A (left panel),1 mm; A (right panel), 100 µm

Fibroblasts are the main cellular components of gum tissue that have an important role in mucosal wound healing [15]. Therefore, to compare the effects of different drugs on the healing ability of oral mucosa, gingival fibroblasts were used to conduct a series of tests. The cellular experiment results showed that the anti-angiogenic drug had a significant inhibitory effect on gingival fibroblasts. Compared with the control group, Suti group had a stronger inhibitory effect on the colony formation of gingival fibroblasts than Zole group, and the combination of zoledronate and sunitinib significantly enhanced the inhibitory effect (*P* < 0.01) (Fig. 4A, B).

**Fig. 5:**
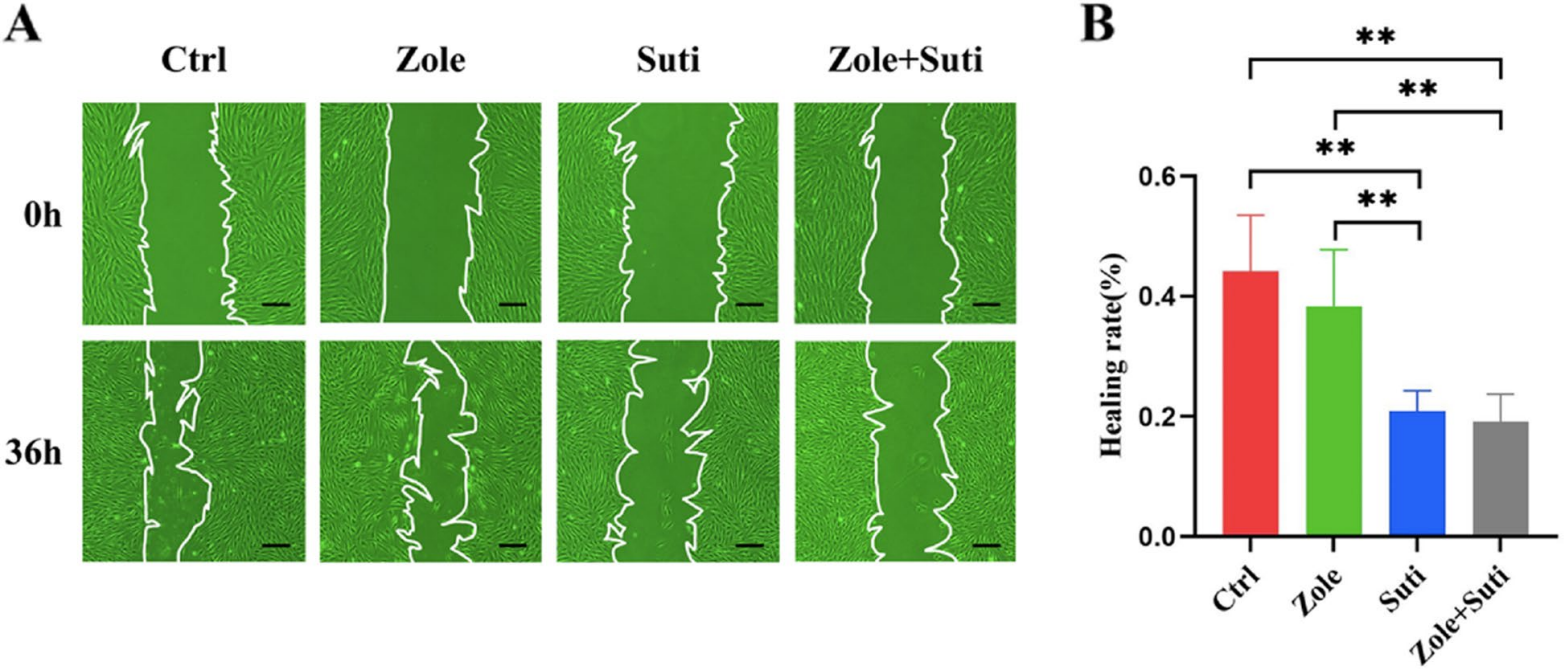
Anti-angiogenic drugs significantly inhibit the migration ability of gingival fibroblasts than anti-resorptive drugs. **A** Microscope observation of scratch wound healing of gingival fibroblasts to observe the migration ability. **B** Statistical analysis of wound healing of gingival fibroblasts 36 h post different drug treatments. ANOVA was performed. *P < 0.05; **P < 0.01. Scale bars in A, 100 µm

**Fig. 6:**
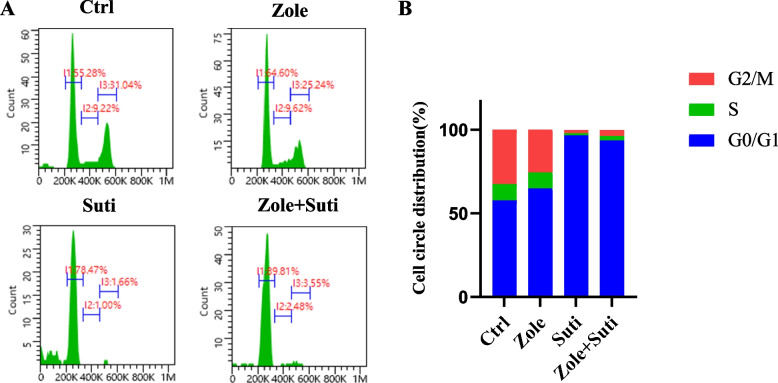
Anti-angiogenic drugs significantly interfere with cell circle activity of gingival fibroblasts than anti-resorptive drugs. **A** Flow cytometry of gingival fibroblasts after PI staining to observe the cell circle distribution. **B** Data summary of cell circle distribution of gingival fibroblasts 48 h after different drug treatments

Next, the CCK-8 assay was used to verify the anti-angiogenic drug's inhibitory effect on gingival fibroblasts during the proliferative procedure, which was consistent with the clone formation test (Fig. 4C). Finally, in order to judge the effect of anti-angiogenic drugs on the migration ability of gingival fibroblasts, a cell scratch test was carried out. The inhibitory effect of anti-angiogenic drugs on fibroblast migration ability was stronger than that of anti-resorptive drugs (*P* < 0.01), and the inhibitory effect of anti-angiogenic was not significantly enhanced after the combined drug treatment (Fig. 5). Finally, through cell cycle analysis, it was found that fibroblasts were mainly blocked in the G0/G1 phase by sunitinib, so the cells could not complete their proliferation activities (Fig. 6).

## Discussion

MRONJ is defined as necrotic bone that occurs in the maxillofacial region after exposure to either anti-resorptive or anti-angiogenic medications [16]. Bisphosphonates (BPs) and denosumab (DMB) are anti-resorptive drugs that have been shown effective in managing cancer-related conditions resulting from bone metastases in the context of solid tumors and the prevention of osteoporosis-related fractures [17]. BPs and DMB can inhibit angiogenesis in vitro and in vivo.

Animal models demonstrated decreased vascularity in sites of extraction sockets and decreased microvessel numbers during the early and late stages of MRONJ development [18]. However, the incidence of MRONJ in patients on anti-angiogenics alone seems less common than in those taking anti-resorptive medications or those taking both drugs [4, 5, 19]. In the 2022 update of the American Association of Oral and Maxillofacial Surgery’s position paper on MRONJ, the diagnostic criteria have been revised to "current or previous treatment with anti-resorptive therapy alone or in combination with immune modulators or anti-angiogenic medications", which means that anti-resorptive drugs may have an important role in the occurrence of MRONJ [16]. Therefore, exploring the aggravating effect of anti-angiogenic drugs on MRONJ disease in anti-resorptive-treated patients is of utmost importance.

The incidence of odontogenic infections in cancer patients is very common. Previous studies have reported that the proportion of chronic and acute odontogenic infections in the dental assessment of cancer patients before chemotherapy is as high as 79% and 44% [20]. Tooth extraction is usually performed when a hopeless tooth condition appears due to periodontitis, dental caries, trauma, etc. [15]. Periodontitis is one of the most common odontogenic diseases, and the application of anti-resorptive drugs based on periodontitis can significantly increase the occurrence of MRONJs after teeth extraction [21]. The socket healing pattern post-tooth extraction follows a bone healing process with a series of orderly biological events to complement epithelial coverage [22].

Fibroblasts are the main cells of gingival tissue that have an important role in teeth extraction healing [23]. Following the inflammatory stage, granulation-related cytokines and growth factors induce fibroblasts' migration and proliferation into the wound site. After migrating into the provisional wound matrix, fibroblasts proliferate and produce matrix metalloproteinases to degrade the provisional matrix, depositing collagen and extracellular matrix (ECM) components to fill up the wound site [24, 25]. Fibroblasts mainly migrate from the nearby dermis to the wound in response to cytokines and growth factors produced by platelets and macrophages in the wounds [25]. Anti-angiogenic drugs have been reported to inhibit platelet function, affecting the blood clot formation that is used as a scaffold for the migration of different cell players [26, 27]. On the other hand, anti-resorptive exposure has been associated with gingival fibroblast death and delayed wound healing, which could be attributed to an elevated inflammatory response and immune dysfunction [28]. Other studies suggest that local injection of endothelial progenitor cells can elevate the amount of VEGF more easily than injecting mesenchymal stem cells, thus enhancing vascularization, improving epithelial and fibroblast functions, and curing the MRONJ lesion [29]. Our experiments suggested that anti-angiogenic drugs combined with anti-bone resorption drugs could seriously affect the proliferation and migration of fibroblasts, which may be the cause of unsatisfactory granulation tissue formation, eventually affecting alveolar bone healing.

Our in vivo study suggested that the anti-angiogenic drug alone could significantly affect the proliferation and migration of fibroblasts without affecting the formation of new bone in the extraction socket. On the other hand, anti-resorptive drugs significantly interfered with the formation of new alveolar bone. Furthermore, after the combined application of the two drugs, the re-epithelialization above the socket could not be completed, suggesting that anti-angiogenic drugs may impair the migration and proliferation of fibroblasts due to a lack of new alveolar bone support.

This study has some limitations. First, the present study was mainly based on the comparative analysis of disease manifestations and cell phenotypes, while the molecular mechanisms were not explored. Thus, future studies should focus on identifying molecular mechanisms of anti-angiogenic drugs in the pathogenesis of anti-resorptive drug-based MRONJ and providing appropriate solutions.

## Conclusions

Our findings support a synergistic contribution of anti-angiogenic drugs to anti-resorptive drugs-based MRONJ. Importantly, the present study revealed that anti-angiogenic drugs alone do not induce severe MRONJ but aggravate the degree of MRONJ through the enhanced inhibitory function of gingival fibroblasts based on anti-resorptive drugs.

## Supplementary Information

**Additional file 1: Supplementary Table 1.** Group information of experimental animals. **Supplementary Figure 1.** Micro-CT images of the periodontitis model of the left maxillary second molar. **Supplementary Figure 2.** Combined use of anti-resorptive and anti-angiogenic drugs interferes with both soft tissue coverage of extraction sockets and new bone formation in extraction sockets. H&E staining to observe the results of soft tissue coverage and new bone formation in the extraction socket of the left maxillary second molar. The inset shows the boxed region magnified. Yellow arrows indicate the formation of a normal mucosal coverage with typical epithelial basement membrane structure. Red arrows indicate empty bone lacuna. Scale bar in A, 100μm. **Supplementary Figure 3.** There is no significant change in the number of osteoclasts in the extraction sockets after different drug treatments. A, TRAP staining of the extraction sockets after different drug treatments. The inset shows the mature osteoclasts boxed region magnified. B, Quantitation of TRAP-positive osteoclasts in the extraction sockets per section from ten independent samples. Scale bar in A, 100μm.

## Data Availability

All data generated or analysed during this study are included in this published article and its supplementary information files.

## Abbreviations

MRONJ: Medication-related osteonecrosis of the jaw
VEGF: Vascular endothelial growth factors
TKI: Tyrosine kinase inhibitors
TRAP: Tartrate resistant acid phosphatase
PFA: Paraformaldehyde
ROI: Region of interest
HGFs: Human gingival fibroblasts
BPs: Bisphosphonates
DMB: Denosumab
