# Supplementary material for: Anti-angiogenic drug aggravates the degree of anti-resorptive drug-based medication-related osteonecrosis of the jaw by impairing the proliferation and migration function of gingival fibroblasts
Source: BMC Oral Health. 2023 May 27;23:330. doi: 10.1186/s12903-023-03034-7 (PMC10225109; doi:10.1186/s12903-023-03034-7)
Supplement: Supplementary file 1 — Additional file 1: Supplementary Table 1. Group information of experimental animals. Supplementary Figure 1. Micro-CT images of the periodontitis model of the left maxillary second molar. Supplementary Figure 2. Combined use of anti-resorptive and anti-angiogenic drugs interferes with both soft tissue coverage of extraction sockets and new bone formation in extraction sockets. H&E staining to observe the results of soft tissue coverage and new bone formation in the extraction socket of the left maxillary second molar. The inset shows the boxed region magnified. Yellow arrows indicate the formation of a normal mucosal coverage with typical epithelial basement membrane structure. Red arrows indicate empty bone lacuna. Scale bar in A, 100μm. Supplementary Figure 3. There is no significant change in the number of osteoclasts in the extraction sockets after different drug treatments. A, TRAP staining of the extraction sockets after different drug treatments. The inset shows the mature osteoclasts boxed region magnified. B, Quantitation of TRAP-positive osteoclasts in the extraction sockets per section from ten independent samples. Scale bar in A, 100μm. [file 12903_2023_3034_MOESM1_ESM.docx]

**Supplementary Table1. Group information of experimental animals**

| **Group** | **Drug treatments** |
| --- | --- |
| Control (Ctrl) | Saline (ip) + corn oil (po) |
| Zoledronate (Zole) | Zoledronate (ip) + corn oil (po) |
| Sunitinib (Suti) | Sunitinib (po) + saline (ip) |
| Zoledronate+sunitinib (Zole+Suti) | Zoledronate (ip) + sunitinib (po) |

**Supplementary figure**


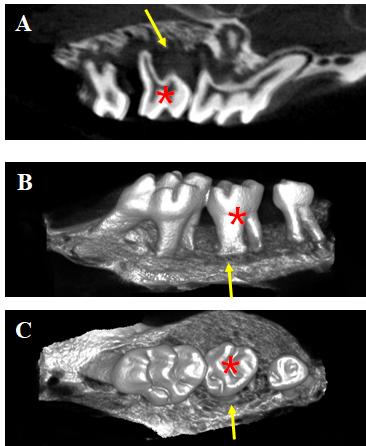


Supplementary Figure 1. Micro-CT images of the periodontitis model of the left maxillary second molar.

A, coronal section of the left maxillary bone and teeth.

B, the palatal side of the 3D reconstruction micro-CT image of the left maxillary alveolar bone and teeth.

C, the occlusal surface of the 3D reconstruction micro-CT image of the left maxillary alveolar bone and teeth.

Asterisks (*) indicate as left maxillary second molar. Yellow arrows（→） shown bone margins after alveolar bone resorption.


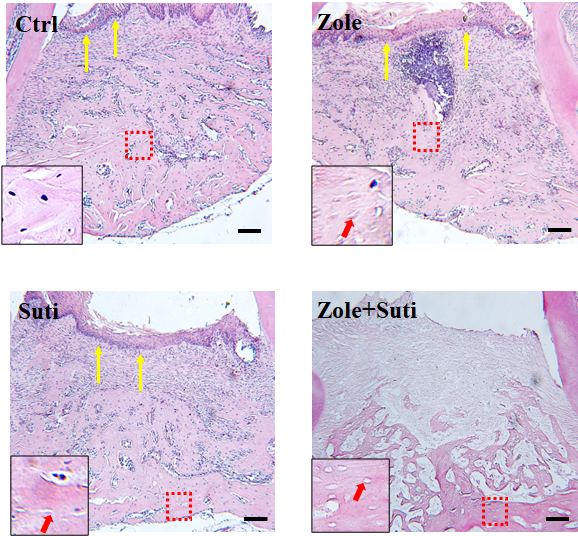


Supplementary Figure 2. Combined use of anti-resorptive and anti-angiogenic drugs interferes with both soft tissue coverage of extraction sockets and new bone formation in extraction sockets. H&E staining to observe the results of soft tissue coverage and new bone formation in the extraction socket of the left maxillary second molar. The inset shows the boxed region magnified. Yellow arrows indicate the formation of a normal mucosal coverage with typical epithelial basement membrane structure. Red arrows indicate empty bone lacuna. Scale bar in A, 100μm.


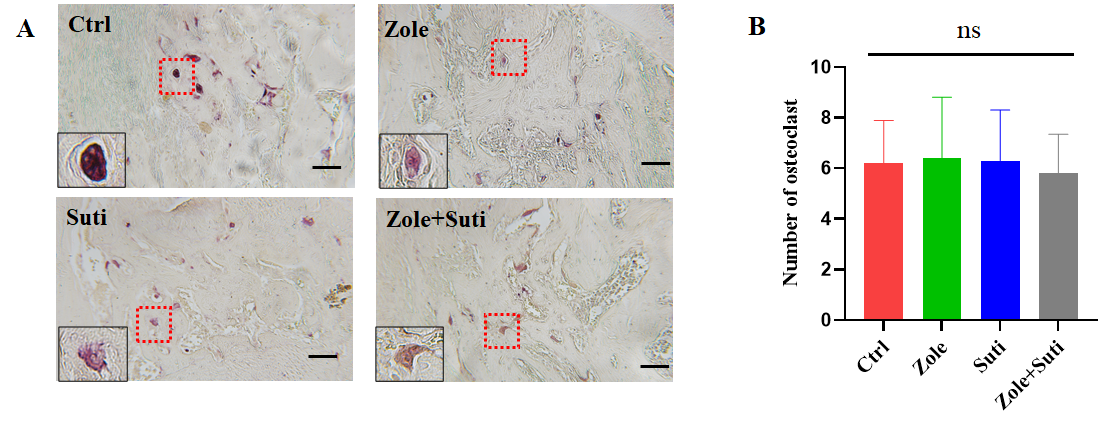


Supplementary Figure 3. There is no significant change in the number of osteoclasts in the extraction sockets after different drug treatments. A, TRAP staining of the extraction sockets after different drug treatments. The inset shows the mature osteoclasts boxed region magnified. B, Quantitation of TRAP-positive osteoclasts in the extraction sockets per section from ten independent samples. Scale bar in A, 100μm.
